# Effects of community-level bed net coverage on malaria morbidity in Lilongwe, Malawi

**DOI:** 10.1186/s12936-017-1767-2

**Published:** 2017-04-07

**Authors:** Veronica Escamilla, Alisa Alker, Leonard Dandalo, Jonathan J. Juliano, William C. Miller, Portia Kamthuza, Tapiwa Tembo, Gerald Tegha, Francis Martinson, Michael Emch, Irving F. Hoffman

**Affiliations:** 1 Carolina Population Center, University of North Carolina, 308 W Rosemary, CB 8120, Chapel Hill, NC 27599 USA; 2 Division of Infectious Diseases, University of North Carolina, Chapel Hill, NC 27599 USA; 3 UNC Project-Malawi, Lilongwe, Malawi; 4 Department of Epidemiology, Gillings School of Global Public Health, University of North Carolina, Chapel Hill, NC 27599 USA; 5 Curriculum in Genetics and Molecular Biology, University of North Carolina, Chapel Hill, NC 27599 USA; 6 Department of Geography, University of North Carolina, Chapel Hill, NC 27599 USA

## Abstract

**Background:**

The protective effect of insecticide-treated bed nets against individual-level malaria transmission is well known, however community-level effects are less understood. Protective effects from community-level bed net use against malaria transmission have been observed in clinical trials, however, the relationship is less clear outside of a controlled research setting. The objective of this research was to investigate the effect of community-level bed net use against malaria transmission outside of a bed net clinical trial setting in Lilongwe, Malawi following national efforts to scale-up ownership of long-lasting, insecticide-treated bed nets.

**Methods:**

An annual, cross-sectional, household-randomized, malaria transmission intensity survey was conducted in Lilongwe, Malawi (2011–2013). Health, demographic, and geographic-location data were collected. Participant blood samples were tested for *Plasmodium falciparum* presence. The percentage of people sleeping under a bed net within 400-m and 1-km radii of all participants was measured. Mixed effects logistic regression models were used to measure the relationship between malaria prevalence and surrounding bed net coverage. Each year, 800 people were enrolled (400 <5 years; 200 5–19 years; 200 ≥20 years; total n = 2400).

**Results:**

From 2011 to 2013, malaria prevalence declined from 12.9 to 5.6%, while bed net use increased from 53.8 to 78.6%. For every 1% increase in community bed net coverage, malaria prevalence decreased among children under 5 years old [adjusted odds ratio: 0.98 (0.96, 1.00)]. Similar effects were observed in participants 5–19 years [unadjusted odds ratio: 0.98 (0.97, 1.00)]; the effect was attenuated after adjusting for individual-level bed net use. Community coverage was not associated with malaria prevalence among adults ≥20 years. Supplemental analyses identified more pronounced indirect protective effects from community-level bed net use against malaria transmission among children under 5 years who were sleeping under a bed net [adjusted odds ratio: 0.97 (0.94, 0.99)], compared to children who were not sleeping under a bed net [adjusted odds ratio: 0.99 (0.97, 1.01)].

**Conclusions:**

Malawi’s efforts to scale up ownership of long-lasting, insecticide-treated bed nets are effective in increasing reported use. Increased community-level bed net coverage appears to provide additional protection against malaria transmission beyond individual use in a real-world context.

**Electronic supplementary material:**

The online version of this article (doi:10.1186/s12936-017-1767-2) contains supplementary material, which is available to authorized users.

## Background

Insecticide-treated bed nets (ITNs) are an important part of the effort to control and eliminate malaria. In previous randomized controlled trials, ITNs decreased both the prevalence of malaria and all-cause mortality in children under 5 years old [1]. However, the effectiveness of ITNs outside the context of clinical trials, where coverage rates tend to be lower, is less clear.

In Malawi, malaria control efforts have focused on scaling up ownership and use of ITNs and long-lasting, insecticide-treated bed nets (LLINs) that do not require retreatment [2]. ITNs are distributed at clinics as part of prenatal care, and periodically through mass distribution campaigns [2–4]. In 2008, the national policy for bed net distribution was amended to include the distribution of LLINs to children under 5 years old attending health facilities [2]. The use of bed nets in Malawi increased: any type of net use increased from 42 to 67% between 2004 and 2010 [5]. But the impact on *Plasmodium falciparum* prevalence is unclear, with a decrease [6, 7] and no change reported [8, 9]. The effectiveness of bed nets likely depends on several factors, including age, the number of children sleeping under one net, the types of mosquitoes and their biting habits in a particular area, how often the bed nets are used, and if the bed nets are kept in good repair [10].

Bed net usage in a community may affect a person’s risk of malaria, regardless of individual use [11–15], as mosquito contact with insecticide treated nets can reduce vector population survival and abundance [16]. However, the rate of community bed net use and the scale at which community coverage influences malaria prevalence are less clear. In addition, community-level bed net effects are commonly examined in clinical trial [15, 17, 18] or modelling scenarios [19], but are less understood outside of a bed net intervention trial setting. The purpose of this study is to explore the relationship between community bed net coverage and malaria prevalence outside of a bed net clinical trial setting following national efforts to scale-up ownership of long-lasting, insecticide-treated bed nets [2, 4].

## Methods

### Study design and data collection

The malaria transmission intensity (MTI) study was an annual cross-sectional survey of *P. falciparum* prevalence, conducted between 2011 and 2013, in support of a multi-site Phase III trial (GSK MAL 055) of the candidate vaccine RTS,S/AS01 [20]. The catchment area comprised Traditional Authorities (TAs) surrounding the Malawi Ministry of Health’s Area 18 health centre. The survey was implemented during the peak malaria season (February–June). Each year, 400 households were randomly selected from a geographic information system (GIS) database containing global positioning system (GPS) coordinates for all catchment area households [21]. Households with a child under 5 years old were eligible to participate. A child under 5 years old was recruited from each household. A second participant over 5 years old was also recruited from each household. Recruitment of older participants included individuals aged 5–19 years, and adults over 20 years, and was alternated by household. A coin toss was used for selection in instances where multiple people in a household of the same age group were eligible to participate. A total of 800 individuals (400 <5 years, 200 5–19 years, 200 >20 years) were recruited annually. Subjects in the RTS,S vaccine trial were excluded from participation.

Field data collection was conducted in teams of three, which included two community health workers, and one nurse. Community health workers were trained to administer questionnaires and collect GPS coordinates of participating households. Field nurses were trained to administer a rapid diagnostic test (RDT) using SD Bioline HRP-2 Kits (Gyeonggi-do, Republic of Korea). The questionnaire, administered by the community health workers, captured demographic, health and behavioural information, including individual-level bed net use the previous night, household mosquito repellent use during the previous 7 days, and household indoor residual spraying during the previous 12 months. Participants were also asked to report the number of household members using a bed net while sleeping. Household characteristics, including wall, floor, roof, window material, and electricity, were also recorded. Household GPS coordinates were linked to each participant.

During the interview, a field nurse conducted a finger prick and collected blood from each participant for a rapid diagnostic test (RDT) and microscopy. The RDT was performed to test participants for the presence of malaria parasitaemia in the field. A field nurse assessed the RDT and clinical status of a participant and provided immediate care and treatment in accordance with national guidelines, and referred the participant to the nearest health facility for treatment. Thick and thin blood smears were prepared. Slides were made in duplicate, and transported to the Area 18 health centre laboratory. For each slide, the presence of parasitaemia was determined independently by two trained microscopists. In the case of non-concordance, parasitaemia was determined by a third microscopist.

ITNs were distributed to all participating households after completion of each annual survey.

### Variable construction

The primary outcome for this study was *P. falciparum* infection represented as a binary variable defined as the presence of any parasitaemia by light microscopy. An index variable representing household wealth was constructed using principal components analysis, and is a composite score of household characteristics including wall, floor, roof, window material, and electricity [22]. The composite wealth index was categorized as low, medium, or high [22].

Using the ‘buffer’ tool in ArcGIS 10.0, 400-m and 1-km radii were placed around each participant household. The intersect tool was used to identify households located within the 400-m and 1-km buffers, from which the total population and the total number of individuals sleeping under a bed net were calculated. The percentage of the study population sleeping under a bed net within each radius, excluding the individual participant was then calculated. Bed net use by close proximity neighbours can reduce individual risk of *P. falciparum* infection [17], and community coverage effects have been identified as far as 1.5-km [12]. Therefore, a 400-m radius was selected because it was the minimum distance required to capture at least one neighbour for each household in the study area. A 1-km radius was selected to measure the effect of community bed net coverage at a larger spatial scale. A 1-km radius captured a large proportion of neighbouring households while maintaining neighbourhood variability between participant households.

Community-level vaccine coverage was also measured to account for potential protective herd effects [23, 24]. Data were obtained from the RTS,S/AS01 candidate malaria vaccine trial to identify the percentage of the population vaccinated in the study area [20]. Using the buffer and intersect tools, community-level vaccine coverage variables were constructed for 400-m and 1-km areas surrounding participant households.

Euclidean distance (i.e., straight line distance) between household and the nearest body of standing water was also measured to capture potential exposure to mosquito breeding areas. Community bed net coverage, vaccine coverage, and distance variables were all constructed using ArcGIS version 10.0 software (Environmental Systems Research Institute, Redlands, USA).

### Data analyses

Sample population characteristics were compared across the three survey periods using Pearson’s Chi square test for categorical variables and the Kruskal–Wallis test for ordinal variables.

Spatiotemporal patterns of malaria prevalence and bed net use were examined using kernel density estimation. A roving window or kernel passes across the study area and computes the density of events within the spatial window. Density estimates are calculated at the window centroid and are based on the weighted values of all events in the window. Events are weighted according to distance from the centroid, with greater weight assigned to events near the centre of the window [25]. Kernel density estimation was selected to visually examine a smoothed study surface while maintaining participant confidentiality. Kernel density estimates were computed and mapped using ArcGIS version 10.0 software (Environmental Systems Research Institute, Redlands, USA).

Community-level bed net coverage (400-m and 1-km) between individuals with malaria and those without malaria was compared using a two-sample t test.

The relationship between community-level bed net coverage and *P. falciparum* infection was examined using mixed effects logistic regression to account for clustering of individuals within each survey period. Models were stratified by age group (<5 years; 5–19 years; ≥20 years). Percentage bed net use among study participants within 400-m and 1-km radii were examined separately. Variables selected a priori included individual ITN use, age, and household wealth. The percentage of vaccinated persons living within 400-m and 1-km of participants were considered at a statistical significance level of p < 0.10. Additional variables considered at a statistical significance level of p < 0.10 included living within 1-km of a river or a large standing body of water, household commercial mosquito repellent use, and indoor residual spraying. Predictive margins were also plotted to examine the probability of *P. falciparum* infection given the percentage bed net coverage within 400-m and 1-km of each household.

### Ethical clearance

This study was approved by the Institutional Review Boards at the University of North Carolina at Chapel Hill (Chapel Hill, North Carolina, USA) and the National Health Sciences Research Committee, Lilongwe, Malawi. Study information was provided in both oral and written form to participants. Written consent was obtained from all participants and legal guardians.

## Results

During 2011–2013, 2400 people (N = 1200 <5 years; n = 600 5–19 years; n = 600 >20 years) were enrolled. Fewer than 1.0% of participants were sampled in multiple periods. Overall, 8.8% (212) of participants tested positive for malaria (Table 1). Malaria prevalence declined each year from 12.9% in 2011 to 8.0% 2012 (p = 0.001), to 5.6% in 2013 (p = 0.059).

**Table 1:** Study population characteristics

	2011	2012	2013	Total	p value
Malaria prevalence by age group, *n* (%)					
<5 years	46 (12.5)	25 (6.3)	14 (3.5)	85 (7.5)	<0.001
5–19 years	38 (19.0)	30 (15.0)	16 (8.0)	84 (14.0)	0.006
>20 years	19 (9.5)	9 (4.5)	15 (7.5)	43 (7.2)	0.149
All age groups	103 (12.9)	64 (8.0)	45 (5.6)	212 (8.8)	<0.001
Individual bed net use by age group, *n* (%)					
<5 years	232 (58.0)	254 (63.5)	330 (82.5)	816 (68.0)	<0.001
5–19 years	86 (43.0)	90 (45.0)	140 (70.0)	316 (52.7)	<0.001
>20 years	112 (56.0)	129 (64.5)	159 (79.5)	400 (66.7)	<0.001
All age groups	430 (53.8)	473 (59.1)	629 (78.6)	1532 (63.8)	<0.001
Household wealth index, *n* (%)					
Low	335 (41.9)	346 (43.3)	296 (37.0)	977 (40.7)	<0.01
Medium	368 (46.0)	346 (43.3)	360 (45.0)	1074 (44.8)	<0.01
High	97 (12.1)	108 (13.5)	144 (18.0)	349 (14.5)	<0.01
Mean percentage of neighbors within a 400-m radius sleeping under bed net, (SD)	58.8 (15.3)				
Mean percentage of neighbors within a 1-km radius sleeping under bed net, (SD)	59.0 (12.0)				

Differences between survey periods measured using Chi square tests for categorical variables and Kruskal–Wallis test for ordinal variables

Overall, 63.8% (N = 1532) of participants reported sleeping under a bed net (Table 1). Of these, 62.8% (N = 962) reported sleeping under an ITN. Malaria prevalence was similar between people sleeping under ITNs and those sleeping under untreated bed nets (5.4 vs 5.8%; p = 0.751). Reported bed net use significantly increased from 53.8% in 2011 to 59.1% in 2012 (p = 0.03), and 78.6% in 2013 (p < 0.001). The largest increase occurred between 2012 and 2013 among children and teenagers aged 5–19 years (45.0 vs 70.0%; p = 0.005).

**Fig. 1:**
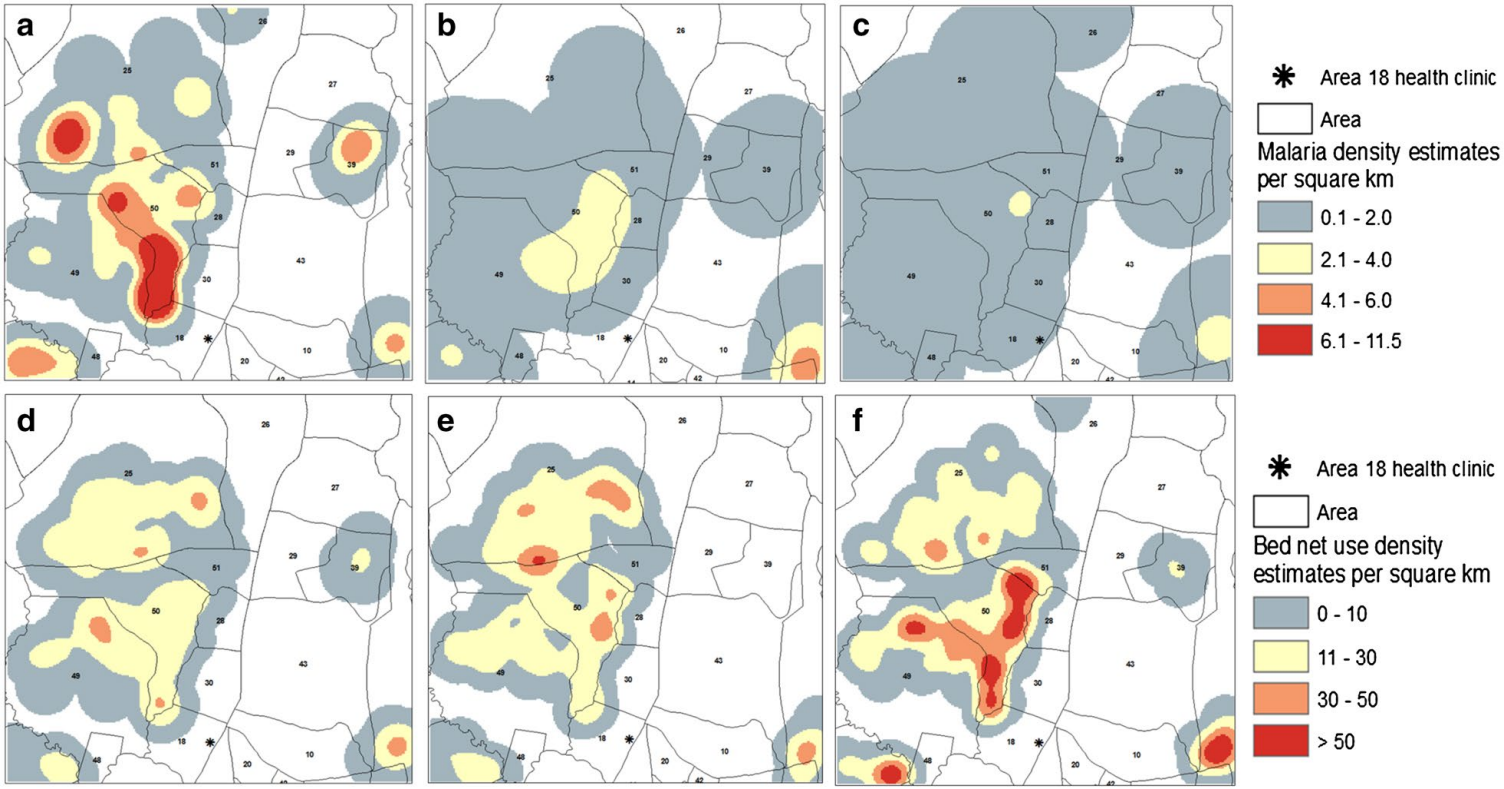
**a**–**c** Kernel density estimates of *Plasmodium falciparum* for each survey period [from *left* 2011–2013]. **d**–**f** Kernel density estimates of reported individual bed net use for each survey period [from *left* 2011–2013]. *Shading* reflects low density estimates in *blue*, and high density estimates in *red*. Malaria prevalence density estimates ranged from 0.1 to 11.5 per sq km. Bed net use density estimates ranged from 0 to >50% of the population per km^2^

The temporal decline in malaria prevalence and increase in bed net use are reflected in the kernel density estimates (Fig. 1). Malaria prevalence density estimates ranged from 0.1 to 11.5 per sq km. Most of the malaria density estimates were <2.0 in 2013, compared to 2011 where several areas had malaria density estimates >4.1, and as high as 11.5 (Fig. 1a, c). Bed net use density estimates increased in 2013 (Fig. 1f). Several areas had density estimates >50 in 2013 compared to previous years (Fig. 1d–f). Increases in bed net use and declines in malaria prevalence did not occur in the same locations across the study area. However, overlap is noted in area 50 where malaria prevalence declined in parallel with a substantial increase in bed net use during the study period (Fig. 1a, c, d, f).

### Participants under 5 years of age

**Fig. 2:**
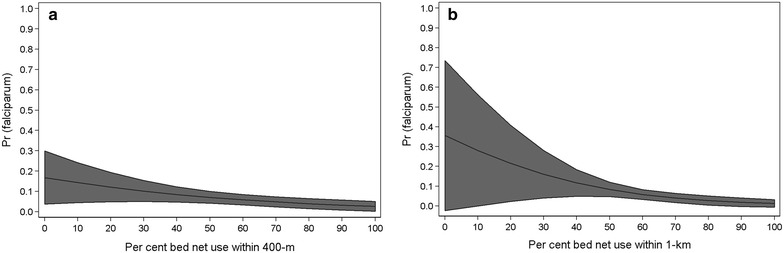
Predicted margins with 95% confidence interval of *Plasmodium falciparum* infection in children <5 years old for per cent increase in bed net coverage within community. **a** Per cent bed net use within 400-m radius. **b** Per cent bed net use within 1-km radius. Community bed net coverage ranges from 10 to 100% as the minimum coverage within 400-m of an individual was 15%

The mean percentage bed net use within a 400-m radius was lower for children with malaria compared to children without malaria (51.4 vs 59.4%, p < 0.001). As expected, malaria prevalence significantly decreased with individual bed net use (unadjusted odds ratio: 0.28, p < 0.01) (Table 2). As the percentage of the population sleeping under a bed net within a 400-m radius increased, the odds of malaria significantly decreased [adjusted odds ratio (AOR): 0.98, 95% confidence interval (CI) 0.96, 1.00] (Table 2). Increased wealth index was protective, and prevalence increased with age (Table 2). The percentage of the population who received the RTS,S malaria vaccine within a 400-m radius was positively associated with malaria prevalence, however the association was no longer significant after adjusting for covariates (Table 2). Average marginal effects indicated a steady decline in the probability of infection as bed net coverage increases within 400-m of a household (Fig. 2).

**Table 2:** Mixed effects logistic regression models stratified by age group

	Unadjusted OR (95% CI)	Adjusted OR (95% CI) 400-m model	Adjusted OR (95% CI) 1-km model
Participants age <5 years (N = 1200)			
Individual sleeps under bed net	0.28 (0.18, 0.45)***	0.44 (0.27, 0.73)***	0.41 (0.25, 0.67)***
Individual does not sleep under bed net	Ref	Ref	Ref
Percent population sleeping under bed net within 400-m	0.96 (0.95, 0.98)***	0.98 (0.96, 1.00)**	–
Percent population sleeping under bed net within 1-km	0.96 (0.94, 0.98)***	–	0.96 (0.93, 0.99)**
Household wealth index			
Low	1.00 (Ref)	1.00 (Ref)	1.00 (Ref)
Medium	0.32 (0.19, 0.53)***	0.40 (0.24, 0.67)***	0.38 (0.23, 0.63)***
High	0.14 (0.04, 0.44)***	0.24 (0.07, 0.79)**	0.21 (0.06, 0.71)**
Age, years	1.28 (1.07, 1.53)***	1.25 (1.04, 1.51)**	1.25 (1.03, 1.51)**
Percent population vaccinated, 400 m	1.04 (1.01, 1.09)***	1.01 (0.97, 1.06)	–
Percent population vaccinated, 1 km	1.03 (1.01, 1.06)***	–	0.95 (0.89, 1.02)
Participants age 5–19 years (N = 600)			
Individual sleeps under bed net	0.42 (0.25, 0.70)***	0.49 (0.29, 0.84)***	0.50 (0.30, 0.85)**
Individual does not sleep under bed net	Ref	Ref	Ref
Percent population sleeping under bed net within 400-m	0.98 (0.97, 1.00)**	0.99 (0.98, 1.01)	–
Percent population sleeping under bed net within 1-km	0.97 (0.95, 0.99)***	–	0.98 (0.96, 1.00)
Household wealth index			
Low	1.00 (Ref)	1.00 (Ref)	1.00 (Ref)
Medium	0.44 (0.26, 0.73)***	0.49 (0.29, 0.83)***	0.50 (0.29, 0.84)**
High	0.48 (0.22, 1.04)*	0.64 (0.29, 1.44)	0.67 (0.30, 1.49)
Age, years	1.00 (0.93, 1.07)	0.99 (0.93, 1.07)	1.00 (0.96, 1.00)
Participants age ≥20 years (N = 600)			
Individual sleeps under bed net	0.68 (0.36, 1.27)	0.66 (0.32, 1.32)	0.7 (0.36, 1.43)
Individual does not sleep under bed net	Ref	Ref	Ref
Percent population sleeping under bed net within 400-m	0.99 (0.97, 1.02)	1.01 (0.99, 1.03)	–
Percent population sleeping under bed net within 1-km	0.98 (0.96, 1.01)	–	1.00 (0.97, 1.03)
Household wealth index			
Low	1.00 (Ref)	1.00 (Ref)	1.00 (Ref)
Medium	0.31 (0.15, 0.64)***	0.34 (0.16, 0.71)***	0.35 (0.17, 0.72)***
High	0.25 (0.09, 0.20)**	0.26 (0.07, 0.91)**	0.29 (0.08, 1.03)*
Age, years	0.89 (0.83, 0.96)***	0.90 (0.84, 0.96)***	0.90 (0.84, 0.96)***

* p < 0.1; ** p < 0.05; *** p < 0.01

Because the effect of individual bed net use was so robust, additional models stratified by individual bed net use were run to further examine the protective effect of community bed net coverage (Additional file 1). The mean percentage of the population sleeping under a bed net within the surrounding 400-m community was significantly higher for children who were sleeping under a bed net compared to children who were not sleeping under a bed net (62.1 vs 51.9%, p < 0.001). Among children sleeping under a bed net (N = 816), malaria prevalence decreased as bed net coverage increased (Additional file 1). Bed net coverage was not associated with malaria prevalence among children under 5 years old who were not sleeping under a bed net (N = 384; Additional file 1). Average marginal effects indicated that the decline in probability of infection as bed net coverage increases is most pronounced for children already sleeping under a bed net (Additional file 2), with a slight decline among children who do not sleep under a bed net (Additional file 2).

The mean percentage of the population sleeping under a bed net within a surrounding 1-km radius was lower for children with malaria compared to children without malaria (54.5 vs 59.0%, p < 0.001). The mixed effects logistic regression results measuring bed net use within 1-km were similar to the 400-m coverage models (Table 2). The odds of malaria decreased as community bed net coverage increased (AOR: 0.96, 95% CI 0.93, 0.99) (Table 2). The percentage of vaccinated individuals living within a 1-km radius was not associated with malaria prevalence in the adjusted model (Table 2). Average marginal effects indicated a steady decline in the probability of infection as bed net coverage increases within 1-km of a household (Fig. 2). Similar results were observed in models stratified by individual bed net use (Additional file 1). Malaria prevalence decreased among children sleeping under a bed net as community-level coverage within a 1-km radius increased (Additional file 1). Community bed net use within 1-km was not associated with malaria prevalence in children under 5 years who did not sleep under a bed net (Additional file 1).

### Participants aged 5–19 years

For participants aged 5–19 years, the mean percentage of the population sleeping under a bed net was lower for those with malaria compared to those without malaria, both within a 400-m radius (55.3 vs 59.8%; p = 0.013) and a 1-km radius (55.9 vs 59.8%; p = 0.006). Individual bed net use had a significant protective effect in both the unadjusted and adjusted models (Table 2). An increase in the percentage of community-level bed net coverage was associated with a decrease in malaria prevalence for both the 400-m and 1-km univariate models (Table 2). This effect was attenuated after adjusting for individual-level bed net use and wealth (Table 2).

### Participants over 20 years old

The mean percentage bed net coverage did not differ between adults with malaria and those without malaria within a 400-m radius (58.5 vs 57.2%; p = 0.581) or a 1-km radius (58.9 vs 56.6%; p = 0.216). In addition, neither individual bed net use nor community-level bed net coverage was associated with malaria (Table 2). Increased wealth index and increased age were protective in adults over 20 years (Table 2).

## Discussion

In this cross-sectional study, a decrease in malaria prevalence among children under 5 years of age as the percentage of the population sleeping under a bed net in the surrounding community increased was observed. Average marginal effects indicated a steep decline in the probability of infection when community coverage reached 50–70% within 400-m and 1-km communities. These findings outside of a bed net clinical trial setting are similar to previous modelling scenarios that found that modest coverage (35–65%) for all adults and children had community-wide benefits [19].

The protective effects of community bed net use were most notable among children under 5 years sleeping under a bed net. While the observed effect size of community-level bed net coverage within 400-m in relation to malaria prevalence in children under 5 years sleeping under a bed net was small (AOR 0.97; Additional file 1: Table S1), reduced odds of 3% for each additional per cent in community bed net use could provide considerable protection. Children not sleeping under bed nets tended to live in communities with lower bed net use. It is possible that the lack of association between community bed net coverage within 400-m and malaria prevalence in children under 5 years who were not sleeping under a bed net was due to a higher vector population in low bed net use communities [16]. The protective effect of community-level bed net use against malaria prevalence in older children (5–19 years) was attenuated after accounting for individual bed net use, possibly in response to a weak community effect, and individual built immunity. The lack of association between community or individual bed net use and malaria prevalence in adults over 20 years could also be due to acquired immunity.

Indirect protective effects from increased community-level bed net coverage have been observed among children under 5 years in previous trial settings [12, 15, 17, 18]. In a trial in western Kenya, similar protective effects were found in compounds located 300-m from compounds using ITNs [17]. Similarly, these findings suggest that the indirect protective effects of community bed net use can be seen in children under 5 years at a 400-m scale. The finding that a community protective effect was observed at a larger 1-km scale supports findings in randomized controlled trials that found community protective effects with coverage spanning 1.5-km [12], and a reduction in mosquito population in villages located 600-m outside of villages receiving an ITN intervention [16].

Similar to previous studies, bed net use was highest among children under 5 years of age, followed by adults over 20 years, with a sharp decline among children and teenagers aged 5–19 years [26]. Bed net use increased across the survey period; the greatest increase occurring between 2012 and 2013 among children and teenagers aged 5–19 years, possibly due to mass bed net distribution campaigns in 2012 [27]. Bed net use measures were based on self-report and could potentially be biased if individuals over-reported. However, the increased bed net use among the 5–19 years age group is similar to findings in Tanzania where previous efforts were made to scale up bed net distribution [28], suggesting that efforts in Malawi are reaching and influencing behaviour among low use populations. Continued efforts to increase bed net use among children and teenagers not only reduces individual malaria but also reduces the reservoir of infection, providing community-wide benefits.

The steep decline in malaria prevalence during the study period is similar to the observed national trend from 2010 (43%) to 2012 (28%) [29]. The pattern suggests a downward trend in malaria transmission, however, longitudinal estimates are needed to confirm this pattern as national prevalence in Malawi increased in 2014 (33%) [29].

It is possible that additional malaria control efforts such as indoor residual spraying occurred simultaneously during the study period and may have influenced the study results, however most control efforts in Malawi focused on bed net distribution [2]. In 2012, Malawi conducted a mass distribution campaign of LLINs, following several years of consistent ITN distribution through the free, clinic-based, routine system [27]. Efforts to achieve universal coverage (one net for every two people) continued beyond 2012 [27]. It was not possible to account for prenatal treatment prevention efforts, but information was collected on household indoor residual spraying, which was very low in the study area.

The study area was located in a vaccine trial catchment area, and it is possible that the presence of the vaccine trial influenced bed net use behavior further impacting mosquito prevalence in the community. It was not possible to measure and evaluate these potential effects for this study. However, the percentage of vaccinated individuals living within 400-m and 1-km radii of study participant households were measured to account for possible protective vaccine herd effects [23, 24]. The positive association between community vaccine coverage and malaria prevalence in children under 5 years in the unadjusted models was unexpected. One possible explanation is that while the RTS,S vaccine trial was individually randomized, study participants were not randomly distributed geographically and potentially lived in higher risk areas with low bed net coverage. The relationship between vaccine coverage and malaria prevalence in children under 5 years was no longer significant in the adjusted models. It is possible that the levels of vaccine coverage were too low to capture additional protective herd effects [23].

The similar prevalence observed between people sleeping under ITNs and those sleeping under untreated bed nets was surprising. These results are inconsistent with previous studies and may be due to inaccuracies in self-reporting of bed net type [30]. Another possibility is that the performance of untreated bed nets may vary geographically and the difference between the individual protective effects of ITNs versus untreated bed nets was not strong enough to detect in the study area.

## Conclusion

The findings of this study suggest that increasing levels of community bed net use can provide added protective benefits beyond individual use. With nearly 80% of the study population sleeping under a bed net in 2013, the results further suggest that Malawi’s efforts to increase bed net use and achieve universal coverage are effective [27]. While bed net coverage has not yet reached 100% in the study area, the added indirect protective effects of increased coverage within the community are already evident.

## Additional files

**Additional file 1.** Mixed effects logistic regression results for children under 5 years stratified by bed net use.

**Additional file 2.** Predicted margins with 95% confidence interval of *Plasmodium falciparum* infection in children <5 years old for per cent increase in bed net coverage within 400-m. **a** Children <5 years who sleep under a bed net; **b** children <5 years who do not sleep under a bed net.
