# Supplementary material for: Effects of community-level bed net coverage on malaria morbidity in Lilongwe, Malawi
Source: Malar J. 2017 Apr 7;16:142. doi: 10.1186/s12936-017-1767-2 (PMC5383956; doi:10.1186/s12936-017-1767-2)
Supplement: Supplementary file 1 — Additional file 1. Mixed effects logistic regression results for children under 5 years stratified by bed net use. [file 12936_2017_1767_MOESM1_ESM.docx]

Additional file 1: Mixed effects logistic regression results for children under 5 years stratified by bed net use

|  |  |  | **Sleep under bed net**  **Adjusted OR (95% CI)**  **(N=816)** |  | **Do not sleep under net**  **Adjusted OR (95% CI)**  **(N=384)** |
| --- | --- | --- | --- | --- | --- |
| Percent population sleeping under bed net within 400-m |  |  | 0.97 (0.94, 0.99)** |  | 0.99 (0.97, 1.01) |
| Percent population sleeping under bed net within 1-km |  |  | 0.94 (0.89, 0.99)** |  | 0.98 (0.94, 1.02) |

All models adjusted for age, wealth index, and percent population vaccinated.

p < 0.1*; p < 0.05**; p < 0.01***
