# Supplementary figures and images for: Effects of community-level bed net coverage on malaria morbidity in Lilongwe, Malawi
Source: Malar J. 2017 Apr 7;16:142. doi: 10.1186/s12936-017-1767-2 (PMC5383956; doi:10.1186/s12936-017-1767-2)

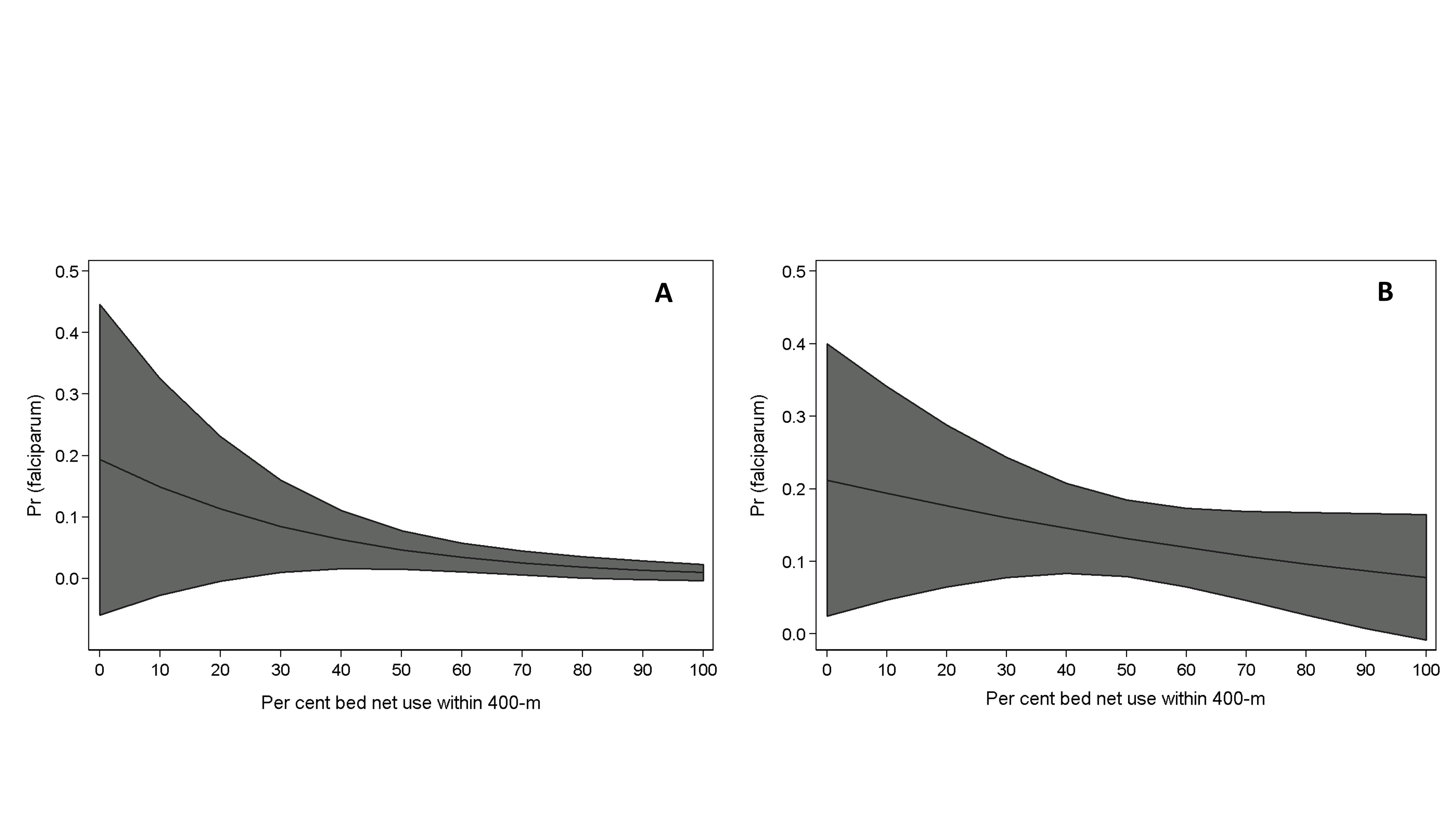

Supplement: Supplementary file 2 — Additional file 2. Predicted margins with 95% confidence interval of Plasmodium falciparum infection in children <5 years old for per cent increase in bed net coverage within 400-m. a Children <5 years who sleep under a bed net; b children <5 years who do not sleep under a bed net. [file 12936_2017_1767_MOESM2_ESM.tif]
